# Biomimetic tissue phantoms for neurosurgical near-infrared fluorescence imaging

**DOI:** 10.1117/1.NPh.10.1.015007

**Published:** 2023-03-15

**Authors:** David Burgos, Bennett Blumenkopf, Ali Afshari, Kirstie Snodderly, T. Joshua Pfefer

**Affiliations:** Food and Drug Administration, Center for Devices and Radiological Health, Silver Spring, Maryland, United States

**Keywords:** aneurysm, phantom, three-dimensional printing, fluorescence

## Abstract

**Significance:**

Neurosurgical fluorescence imaging is a well-established clinical approach with a growing range of indications for use. However, this technology lacks effective phantom-based tools for development, performance testing, and clinician training.

**Aim:**

Our primary aim was to develop and evaluate 3D-printed phantoms capable of optically and morphologically simulating neurovasculature under fluorescence angiography.

**Approach:**

Volumetric digital maps of the circle of Willis with basilar and posterior communicator artery aneurysms, along with surrounding cerebral tissue, were generated. Phantoms were fabricated with a stereolithography printer using custom photopolymer composites, then visualized under white light and near-infrared fluorescence imaging.

**Results:**

Feature sizes of printed components were found to be within 13% of digital models. Phantoms exhibited realistic optical properties and convincingly recapitulated fluorescence angiography scenes.

**Conclusions:**

Methods identified in this study can facilitate the development of realistic phantoms as powerful new tools for fluorescence imaging.

## 1 Introduction

Intracranial saccular aneurysms are localized dilations of a cerebral artery as a result of vessel wall weakening. In the United States, up to 33,000 people each year are affected by hemorrhaging from ruptured cerebral aneurysms, which often results in neurological deficits.^1^ Of the greater than 120,000 patients treated for unruptured cerebral aneurysms between 2004 and 2014, 39% were treated with surgical clipping.^2^ Aneurysms appear most commonly in the circle of Willis, a set of arteries at the base of the brain that direct blood from the heart to the different lobes of the brain. Saccular aneurysms consist of a neck and a dome; identifying the neck is critical to the clipping procedure. Indocyanine green (ICG) is a clinical near infrared (NIR) fluorescent dye administered intravenously to enhance visualization of vascular structures in a range of neurosurgical procedures. Surgeons use standalone or integrated surgical microscopes with an NIR imaging setting to view ICG. Recommended dosage of ICG for angiography is between 0.2 and 0.5  mg/kg of body mass.^3^ In surgical clipping procedures, intraoperative video angiography is used to locate the aneurysm neck and determine whether the surrounding arteries remain patent after the clip is placed.^4^^,^^5^ After clipping, ICG angiography is also used to determine that the neck is fully sealed.^6^

As with any medical imaging system, ensuring performance is essential to clinical outcomes. This is typically accomplished using test methods involving targets and tissue-simulating phantoms described in international consensus standards.^7^ However, such standards do not currently exist for fluorescence imagers. Fluorescence imaging phantoms with simple geometries have been developed for evaluating spatial resolution, depth of field, and other fundamental characteristics.^8^^–^^11^ Our group pioneered the use of three-dimensional (3D) printing to fabricate phantom-based tools for evaluation of biophotonic imaging systems^12^ and, along with several other groups, have made great strides in this area. We have applied molding and 3D printing approaches to develop simple geometry test target phantoms for fluorescence imaging^8^ and collaborated on the development of phantoms doped with IR-125 fluorescent dye to evaluate commercially available clinical imagers.^13^

Research on other medical imaging modalities has demonstrated the utility of realistic phantoms to assess imaging system capability in measuring changes in size of malignant tumors.^14^^,^^15^ Biomimetic fluorescent phantoms can be used for quantitative evaluation of image quality (e.g., size measurement accuracy), or as a way to qualitatively assess how device design modifications that cause trade-offs in image quality (e.g., resolution vs. depth of field) impact visualization of a true surgical scene. Using 3D printing, we have developed biomimetic vascular phantoms derived from 2D and 3D clinical images of the retina^16^ and forebrain,^17^ respectively. These studies included development of methods for fabricating, cleaning and validating complex phantoms with tortuous vessels. Significant advances by other groups have included the development of complex 3D-printed phantoms incorporating near-IR absorbers to achieve specific optical property targets^18^ and the fabrication of phantoms for key NIR imaging applications, such as *in vivo* fluorescence detection of rheumatoid diseases^19^ and cancer biomarkers in solid tumors.^20^ 3D-printed tissue phantoms have also been developed that mimic small animals for diffuse optical and fluorescence imaging studies.^21^^,^^22^ It is also worth noting that fluorescent phantoms have potential as surgical training tools.^23^^,^^24^ However, we are not aware of prior studies addressing biorealistic, image-derived phantoms with fluorescent components as surgical training and planning tools.^25^

The purpose of this study was to investigate the potential for using 3D printing to fabricate biomimetic, image-derived fluorescence phantoms as clinically relevant performance testing tools for neurovascular applications. Primary goals included designing, fabricating, characterizing and implementing a phantom that enables visual simulation of fluorescence angiography during surgical procedures for intracranial aneurysms.

## 2 Methods

Our approach involved three main phases: a design phase in which a 3D digital model of an aneurysm was acquired and incorporated into a larger cerebral model; a fabrication phase involving development of materials and best practices as well as the actual 3D printing, post-processing and assembly of the components; and an evaluation phase where the final models were assessed with an NIR fluorescence imaging system and other approaches.

### 2.1 Generation of Digital Model

**Fig. 1:**
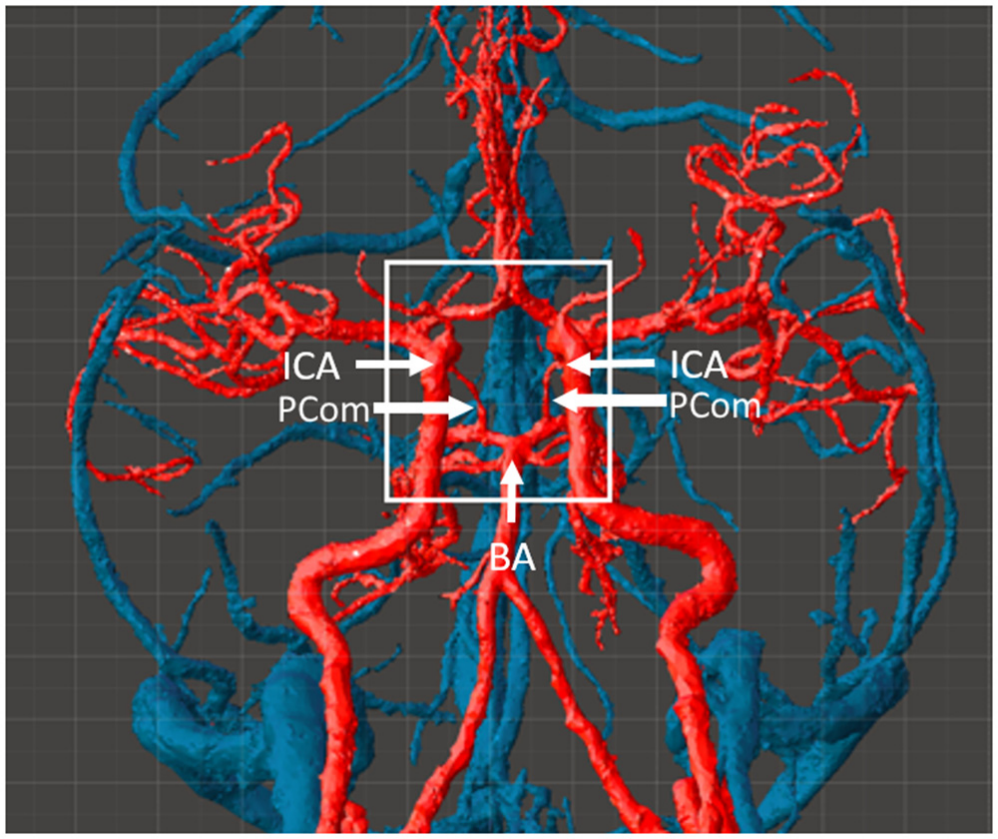
Inferior view of neurovasculature from the MIDA model, with circle of Willis in boxed region. (ICA, internal carotid artery; BA: basilar artery; and PCOM: posterior communicating artery).

**Fig. 2:**
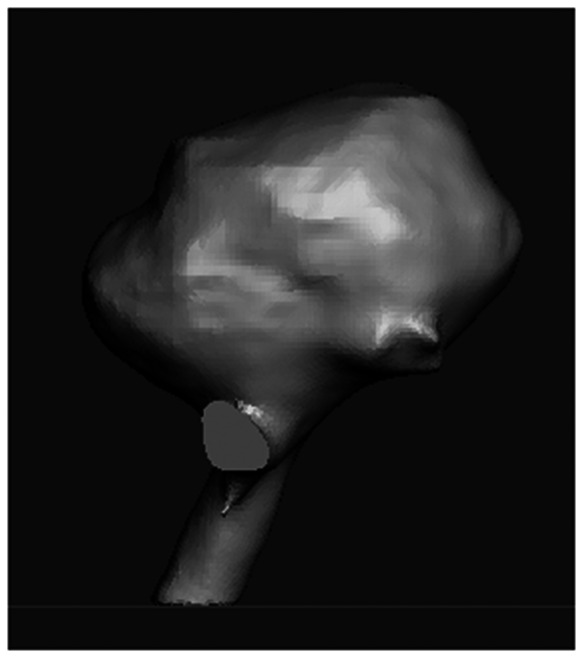
Digital model of aneurysm.^27^

The digital model represents a combination of data from two sources. To generate realistic biological anatomy around the aneurysm, we converted the multimodal imaging-based detailed anatomical (MIDA) model of the head and neck^26^ to an STL file format (Fig. 1). Tissue types incorporated in our model include cerebral arteries, gray matter of the temporal and frontal lobes, second cranial nerve with optic chiasm and the brain stem. Using 3D modeling software (Meshmixer, Autodesk, San Rafael, California, United States), the models were cropped, and secondary components were removed to isolate the cerebral tissue at the base of the brain surrounding the circle of Willis. An aneurysm was extracted from a 3D digital model of the cerebral vasculature with a low-lying saccular aneurysm at the basilar artery (BA) bifurcation^27^ (Fig. 2). This aneurysm was then added into the MIDA-based model at the basilar bifurcation.

In a second design, we attached the isolated aneurysm to the posterior communicating (PCom) artery. These locations were chosen because they are among the sites in the circle of Willis where aneurysms most frequently form.^28^ We then created a perforator artery on the superior surface of the P1 section of the posterior cerebral artery. Our aim was to have a perforator artery near the neck of the aneurysm to simulate a situation where surgeons must avoid these arteries during a clipping procedure.

To avoid part fractures during support removal in the fabrication phase (post-processing), the diameter of some of the more delicate elements across the printed models were increased. Based on data from the literature we determined the mean diameter of posterior communicator vessels to be 1.5 mm and the standard deviation to be 0.3 mm^29^ and increased the diameter of these vessels to 1.8 mm so as to reduce the likelihood of breakage in post-processing. An iterative process was required to identify a minimum perforator vessel diameter—1.0 mm—that could be printed and remain attached to the model after the support structures were removed.

**Fig. 3:**
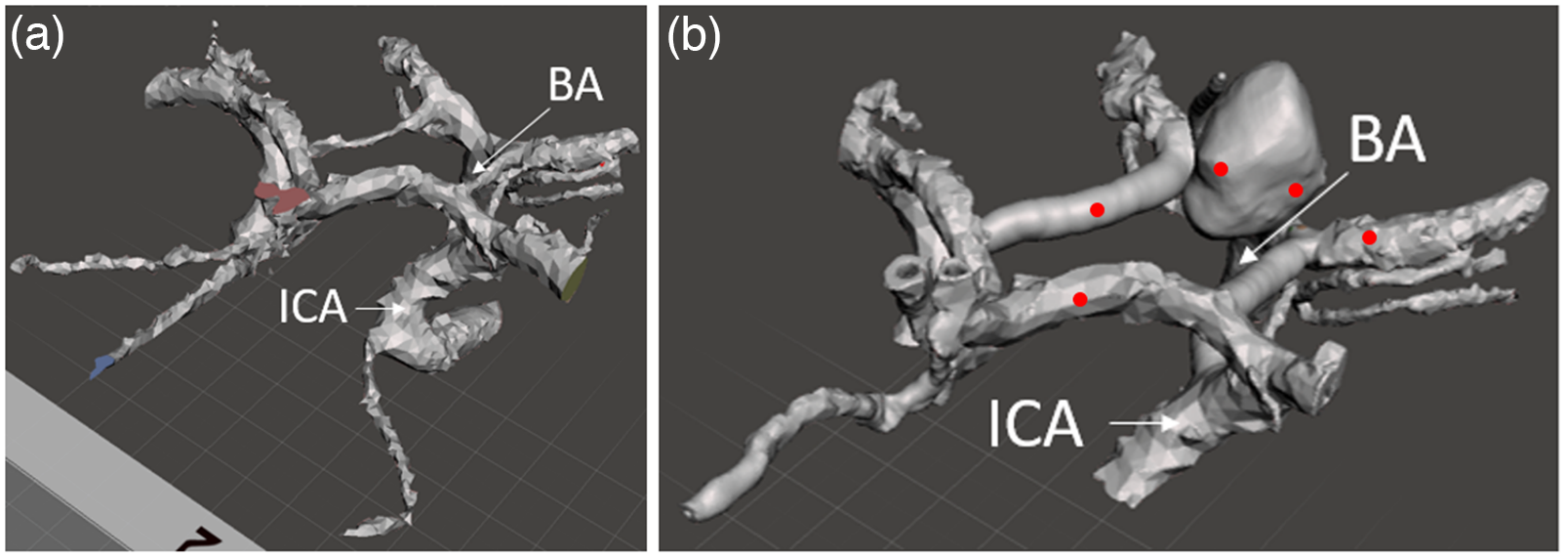
(a) MIDA model exported as STL cropped to the circle of Willis and (b) final digital vascular map including aneurysm. Locations measured with calipers in printed models marked with red dots.

A comparison of initial and final vascular component designs is provided in Fig. 3. Images include the arteries of the MIDA model cropped to the circle of Willis area, as compared to the final digital model of the circle of Willis with a BA aneurysm. In the latter data set, the polygon count was increased, and the vessels were smoothed. The MIDA model also provided the geometry for dura mater, gray matter, brainstem, and optic nerve components. The brain stem and optic nerve model also featured cranial nerves I, II, III, and VI, as these nerves would be visible during some craniotomies. Cranial nerves I and VI were not rooted to the brain tissue within our area of interest. Attempts to print them used thin attachment structures to anchor these cranial nerves to the nearest point on the digital model.

The digital design (STL) files described here have been uploaded to the NIH 3D Print Exchange and are freely available for download at Ref. 30.

### 2.2 Phantom Fabrication with 3D Printer

All phantom components were fabricated with a 3D printer (Form2, Formlabs, Inc., Somerville, Massachusetts, United States) employing a stereolithography approach, which involves optical curing of liquid photopolymers in a layer-by-layer process. The Form2 printer has a nominal minimum feature size of 150  μm in the XY plane, and 35  μm in Z. It was selected due to its relatively low cost and ability to accommodate custom resins formulated by combining multiple proprietary resins and/or adding optical absorbers, scatterers, or fluorophores. This is a somewhat unique feature and one that is very powerful for users requiring flexibility in creating phantoms with well-defined and biologically relevant optical properties.

Using proprietary formulations (Color Kit, Formlabs, Inc., Somerville, Massachusetts, United States) we created a fluorescent photopolymer resin by combining the color kit base material and black resin dye with IR 125 (Exciton, Lockbourne, Ohio, United States) fluorescent dye. IR 125 dye provided excitation and emission properties similar to ICG.^13^ The concentration of black dye was adjusted so that the resin would approximate the $\mu_{a}$ of oxygenated blood. The optical properties of a given formulation was initially estimated using a ultraviolet (UV) chamber (Formcure, Formlabs, Inc., Somerville, Massachusetts, United States) to cure small samples with different concentrations of black dye, then measuring sample optical properties with a spectrophotometer and inverse adding-doubling software (IAD).^31^ Surrounding (non-vascular) tissue regions were printed using the non-fluorescent custom material, a mixture of proprietary white and clear photopolymer resins (Formlabs, Inc., Somerville, Massachusetts, United States). To verify the optical properties of potentially optimal custom formulations, samples were fabricated using the 3D printer and measured with the spectrophotometer and IAD. An overview of the tissue components used to form a final phantom is provided in Table 1.

**Table 1:** Summary of 3D-printed phantom components.

Component	Digital model (s)	Fluorescent	$\mu_{a}$ at 780 nm (1/cm)	$\mu_{s}^{\prime }$ at 780 nm (1/cm)	$\mu_{a}$ at 825 nm (1/cm)	$\mu_{s}^{\prime }$ at 825 nm (1/cm)
Circle of Willis with BA aneurysm	MIDA and saccular aneurysm	Yes	2.8	16.0	2.6	14.8
Circle of Willis with posterior communicator aneurysm	MIDA and saccular aneurysm	Yes	2.8	16.0	2.6	14.8
Gray matter	MIDA	No	0.4	6.7	0.4	6.3
Dura tent	MIDA	No	0.4	6.7	0.4	6.3
Brainstem and optic nerves	MIDA	No	0.4	6.7	0.4	6.3

When the digital model was ready to be fabricated, it was exported in STL format. The STL file was transferred to the Materialise Magics 24.0 (Materialise, Leuven, Belgium) where the fix wizard was used to check for and resolve any remaining errors in the mesh; the number of triangles was also reduced to allow for better translation to the printer software. The file was oriented within the PreForm software (Formlabs, Inc., Somerville, Massachusetts, United States) at an angle such that the most delicate features contacted the fewest and smallest support structures. Support locations were modified manually to ensure fidelity of critical features.

Post-processing of printed parts involved a 15-min wash in isopropyl alcohol (FormWash, Formlabs, Inc., Somerville, Massachusetts, United States), manual separation of the support structures contacting the most delicate features using flush cutters, UV curing, and removal of the remaining support scaffolding material. All support material removal was carefully completed by hand to minimize damage to small components of the phantoms.

**Fig. 4:**
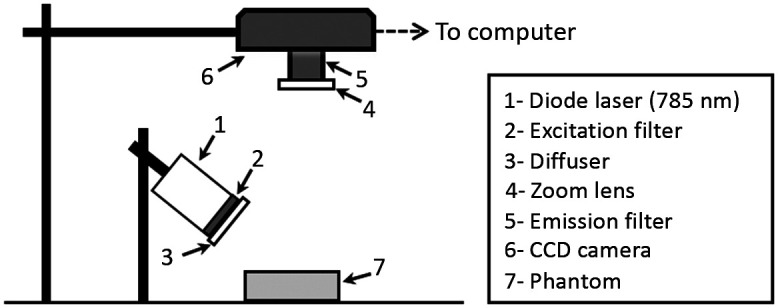
Illustrated layout of the fluorescence imaging system.

Both test samples and vascular phantoms were imaged with a custom NIR fluorescence (NIRF) imaging system. A 785-nm diode laser (RLTMDL-785-1W-5, Lasertechnik, Vienna, Austria) was used along with an 800-nm short-pass excitation filter (84-729, Edmund Optics, Barrington, New Jersey, United States) and a diffuser to illuminate the phantom. Irradiance at the phantom was $2.1\;\mathrm{mW}/{\mathrm{cm}}^{2}$, as measured by a power meter (1918-C and 818-ST-UV, Newport Corp., Irvine, California, United States). Fluorescence emitted by the sample passed through an 825 nm long-pass emission filter (86-078, Edmund Optics, Barrington, New Jersey, United States) and was detected by a 16-bit monochrome CCD camera (1200  pixels×1600  pixels, Alta U2000, Apogee Imaging Systems, Roseville, California, United States) using 100 ms acquisitions. A diagram of the imaging setup is displayed in Fig. 4. Micro-Manager software (version 1.4.20, Univ. of California San Francisco, California, United States) was used for camera control and acquisition, and ImageJ was used to view the images (Fuji, NIH, Bethesda, Maryland, United States).

To validate the models, caliper diameter measurements were taken at three different points along the A1 segment of the right anterior cerebral artery, the left PCom artery, and the P2 segment of the left posterior cerebral artery on two iterations of the printed basilar artery aneurysm phantom. Measurements of the diameter of these vessels were also taken in the digital model at the same locations. These locations are marked with red dots in Fig. 3. From this information we calculated an error level for each of the three vessels. A similar comparison was carried out on the longest diameter of the aneurysm and a second, perpendicular diameter. The mean of the five error levels was used to evaluate the validity of each printed vascular model.

## 3 Results and Discussion

### 3.1 Tissue Mimicking Material (TMM) Development

**Fig. 5:**
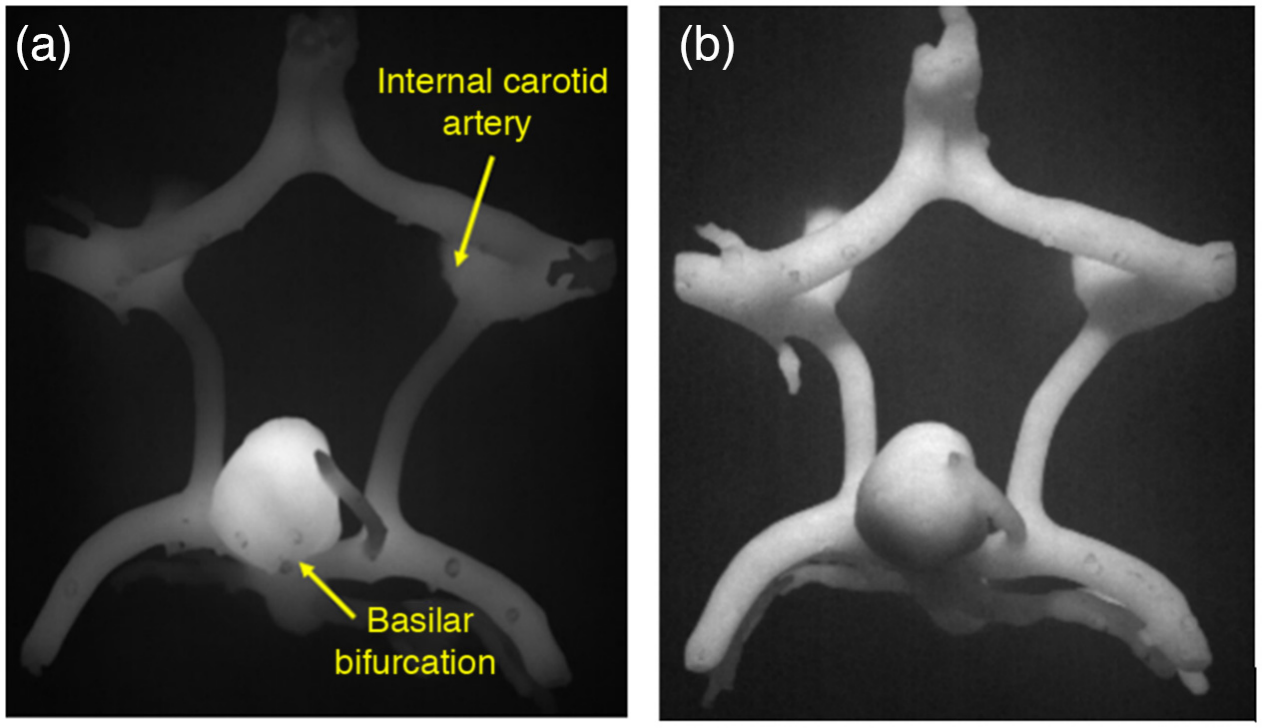
(a) NIRF images of initial version of vascular network component (Fluor V0 material) and (b) final version (Fluor V4 material).

**Fig. 6:**
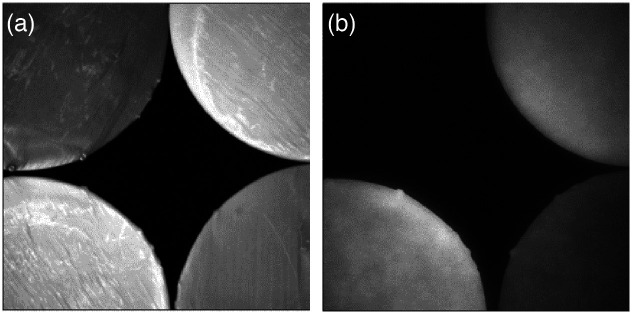
White light (a) and NIRF (b) images of TMM prototypes (clockwise starting with top left: resin mixture V1, V2, V3, and V4-final).

**Fig. 7:**
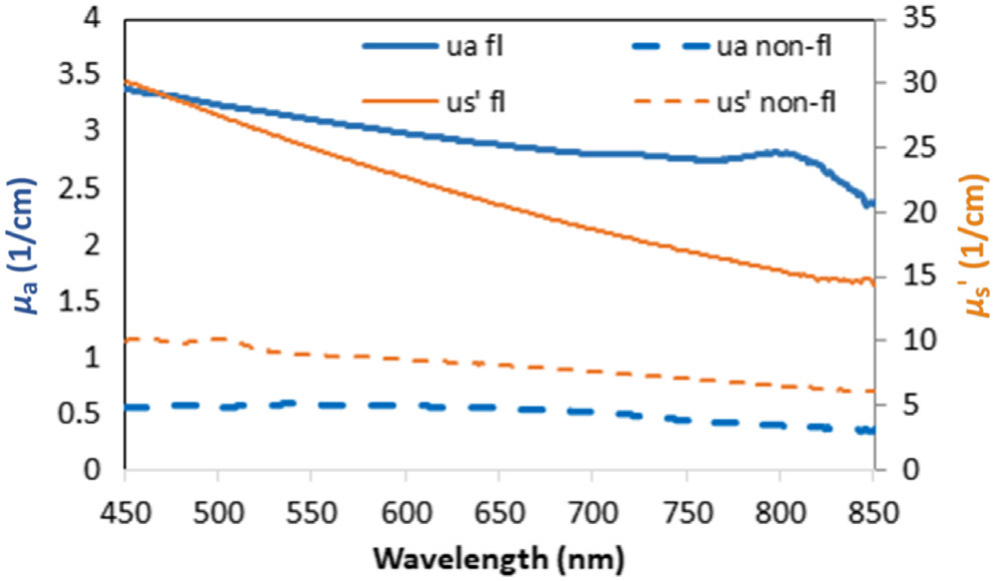
Optical properties (ua, mu_a - absorption coefficient, us’, mu_s’ - reduced scattering coefficient) of final fluorescent (fl), and non-fluorescent (non-fl) custom 3D-printed TMMs.

Vascular phantom components were initially printed with a composite material consisting of the 75% clear resin and 25% white resin by volume (material version V0). Early versions of the custom fluorescent resin contained 300 nM IR-125 in dimethyl sulfoxide (DMSO) (V0) and produced phantoms showing a strong correlation between local structure volume and NIRF signal intensity (i.e., larger features displayed greater signal intensity), as seen in Fig. 5(a)—which is not typical of clinical images. When the $\mu_{a}$ of the vasculature was increased to a more biologically relevant range through the addition of black dye, this size-dependent effect was no longer present. This is notable in part because phantom studies do not always describe careful targeting of fluorescent inclusion absorption coefficient.^21^^,^^23^^,^^24^ For the final series of custom fluorescent materials (V1 to V4), we used the proprietary color kit base and black dye (Formlabs, Inc.) mixed with the IR-125/DMSO solution (Fig. 6). The final formulation involved a 0.75% concentration of black dye by volume and an IR-125 concentration of 1.0  μM, which is consistent with prior fluorescent phantoms.^13^ At this level of IR-125, the signal intensity of cured resin in the NIRF images provided much more clear delineation between fluorescent and non-fluorescent structures. The final $\mu_{a}$ was $2.8\;{\mathrm{cm}}^{-1}$ at a wavelength of 780 nm, which is 15% less than the $\mu_{a}$ of oxygenated blood at the same wavelength reported previously.^32^ The final ${\mu_{s}}^{\prime }$ was $16.0\;{\mathrm{cm}}^{-1}$ at a wavelength of 780 nm, which is about 5.9% lower than the target value for blood, as reported by Bosschaart et al.^32^ Figure 7 shows how the optical properties of the custom fluorescent resin compared with the custom non-fluorescent resin. The final fluorescent resin also exhibits a highly uniform—and thus clinically realistic—intensity distribution in fluorescence images [Fig. 5(b)]. The non-fluorescent phantoms are printed from a mixture of 75% clear resin and 25% white resin (Formlabs, Inc.) by volume. All phantoms were stored at room temperature and no special enclosure was employed. The vascular phantoms are especially delicate, and careful handling is advised.

**Fig. 8:**
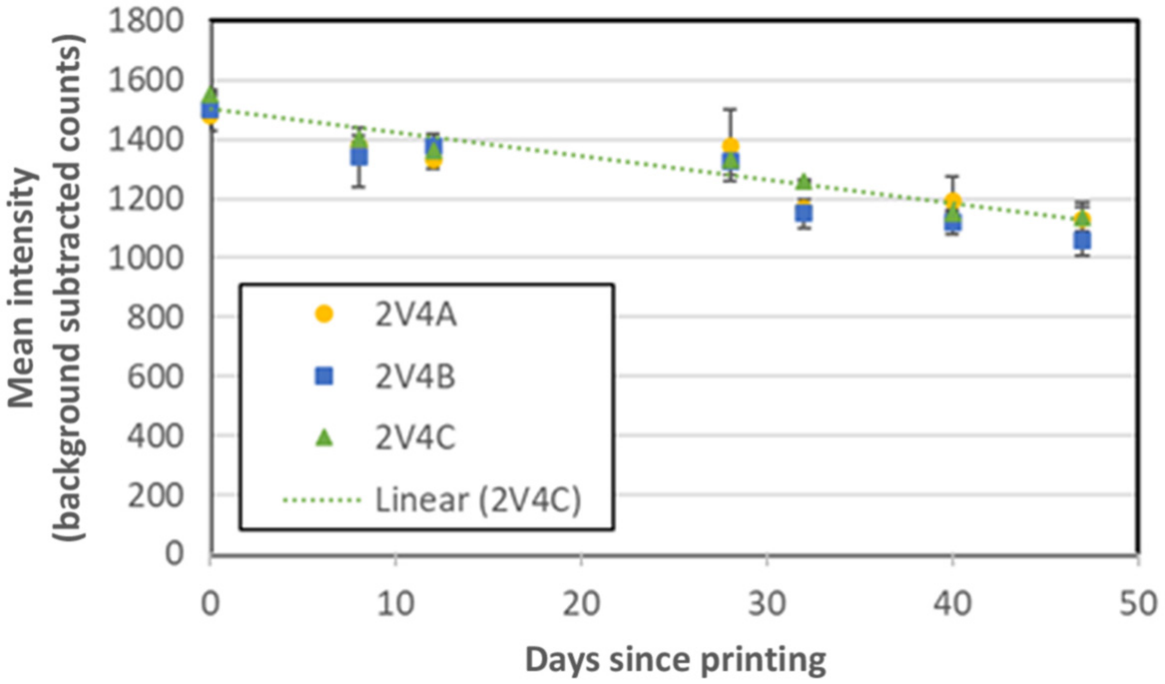
Stability of TMM fluorescence intensity. In the key the first number is the batch number. The second number that follows the letter V is the version number. The last letter indicates which disk in the batch is being referenced. For example 2V4A refers to “batch 2, resin version 4, disk A”.

The stability of the printed fluorescent models was evaluated by measuring the signal intensity, absorption coefficient and reduced scattering coefficient of sample disks over the course of six weeks. A gradual decrease in fluorescence intensity was seen, leading to an overall 26% decrease in signal over 46 days (Fig. 8). These results indicate that while these phantoms should be suitable for use in a variety of applications over a period of a few weeks, additional work is needed to improve stability—especially for applications involving quantitative measurements of fluorescence intensity. Differences between the photostability of our current phantoms and a prior fluorescence phantoms based on polyurethane doped with IR-125 may be due to the use of a different matrix material as well as UV exposure by the 3D printer. Negligible changes in $\mu_{a}$ and ${\mu_{s}}^{\prime }$ were seen over the course of the aforementioned 6-week study. We have also evaluated changes in optical properties over 2 years in Formlab’s white resin; results indicated an increase in $\mu_{a}$ of less than $0.02\;{\mathrm{cm}}^{-1}$ (32%) and a decrease in ${\mu_{s}}^{\prime }$ of $<0.2\;{\mathrm{cm}}^{-1}$ (7%) at 800 nm. Such long-term variations in optical properties indicate that there may be room for improvement in storage approaches and/or 3D printing composite formulations that enhance the ability of these tools to assess temporal changes in device performance.

### 3.2 Geometry

**Fig. 9:**
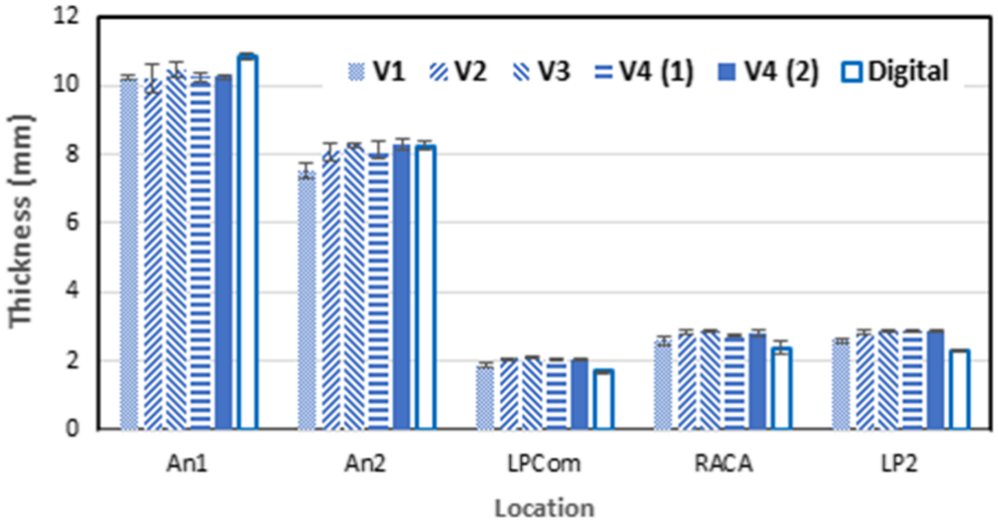
Component thickness measurements across several printed versions, as compared to the digital model of the BA aneurysm phantoms. An, aneurysm diameter; LPCom, left PCom artery diameter; RACA, right anterior cerebral artery diameter; and LP2, P2 segment of posterior cerebral artery diameter. Error bars calculated via standard deviation in Microsoft Excel.

Posterior communicating arteries were printed with a diameter of 1.8 mm to ensure mechanical integrity; this value is, within one standard deviation of the true diameter.^29^ When caliper measurements were compared to dimensions from the digital model, the mean error in spatial features of the BA aneurysm model was determined to be 13.0%. The results of these size validation measurements for BA-aneurysm-model phantoms using different versions of the custom resin can be seen in Fig. 9. Discrepancies on the order of 0.1 mm between the digital model and final printed versions shown here are similar to those found in previous studies with the same printer,^33^^,^^34^ indicating that the nominal resolution of the printer overestimates the accuracy of the final printed samples. The ability to fabricate smaller vascular features would likely improve the realism of the model, however, fine vasculature would also be very fragile. Thus, in the future, we intend to study alternate materials that provide greater flexibility and mechanical strength as well as approaches for achieving improved printing resolution.

**Fig. 10:**
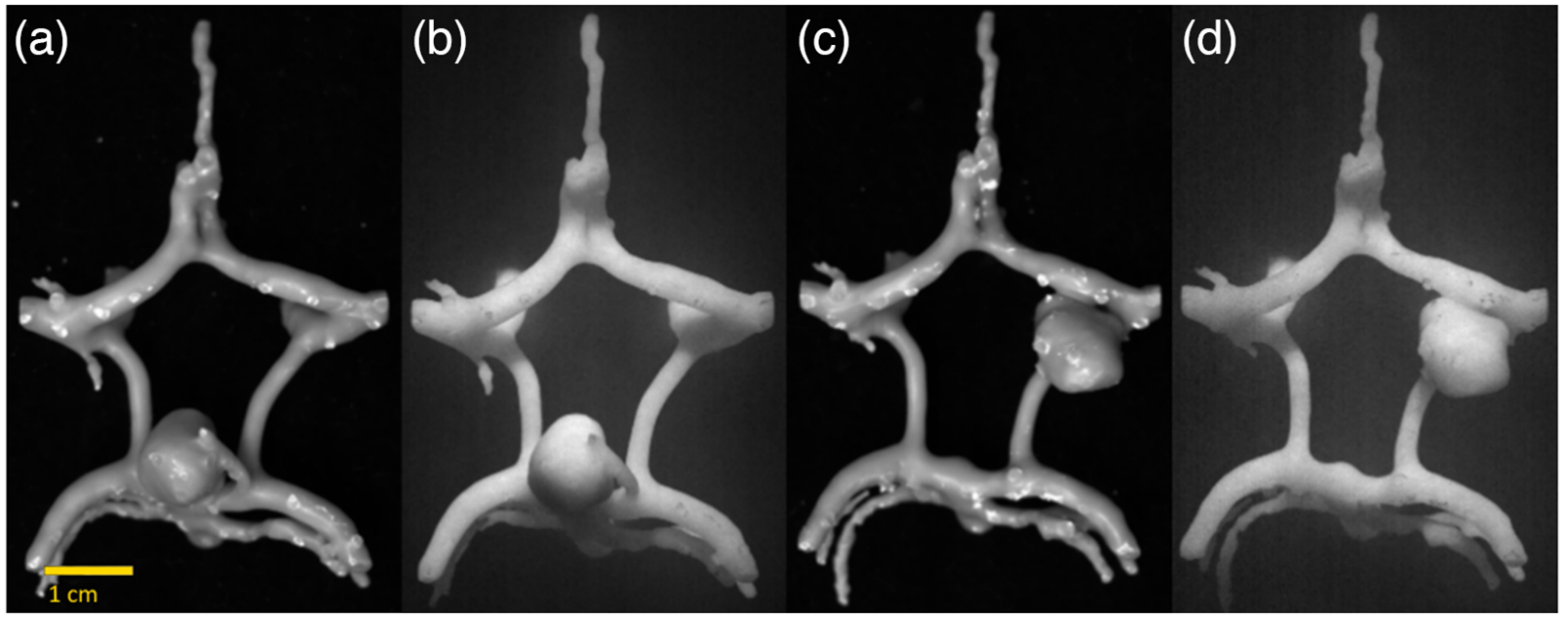
White light (a, c) and NIRF images (b, d) of the circle of Willis component incorporating (a, b) a BA aneurysm and (c, d) a posterior communicator aneurysm.

**Fig. 11:**
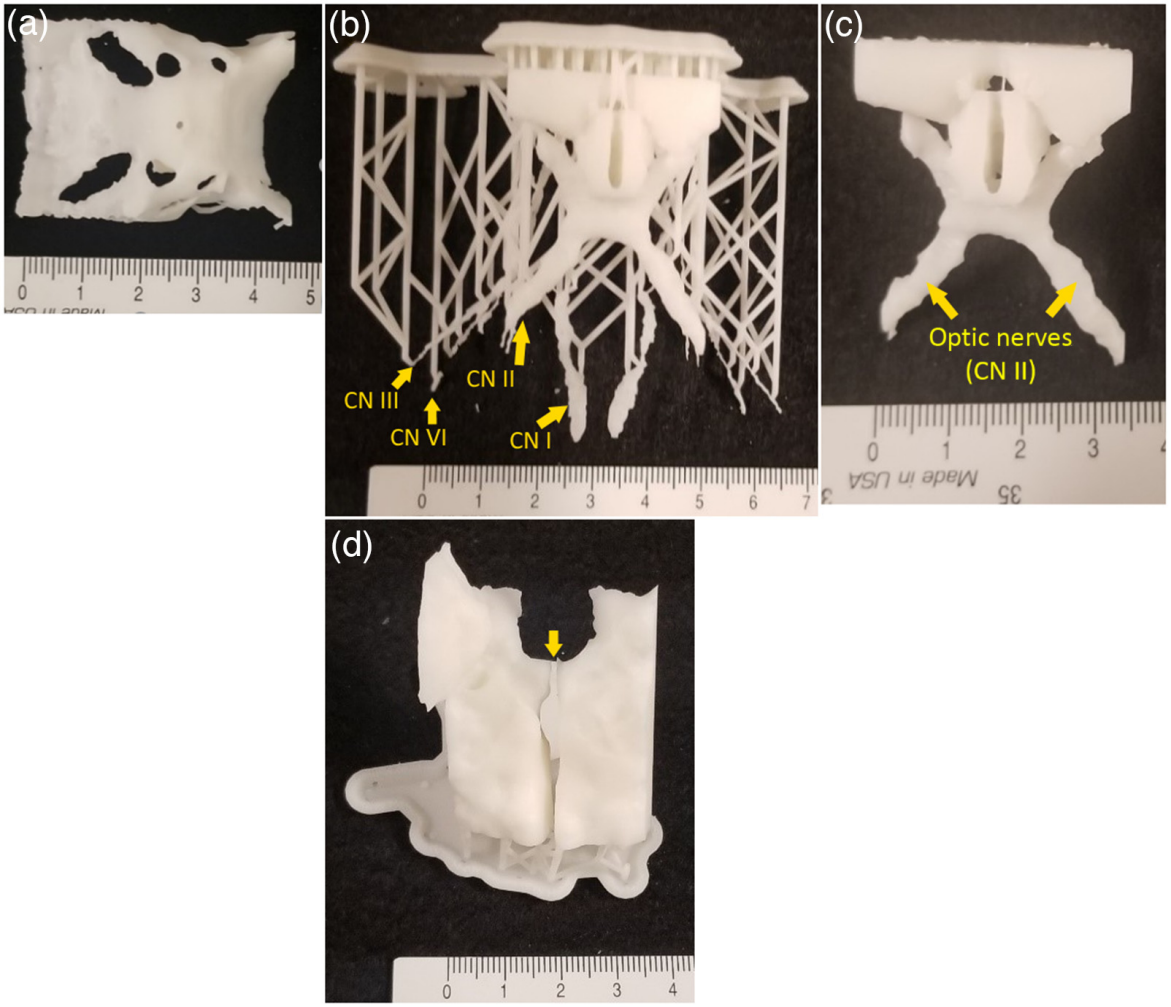
Dura tent model (a), and cranial nerves and brainstem before (b) and after (c) removal of support materal (CN – cranial nerve). Gray matter model before support material removal (d) 1 mm is the shortest space between the two lobes (a yellow arrow shows this space between the two lobes).

**Fig. 12:**
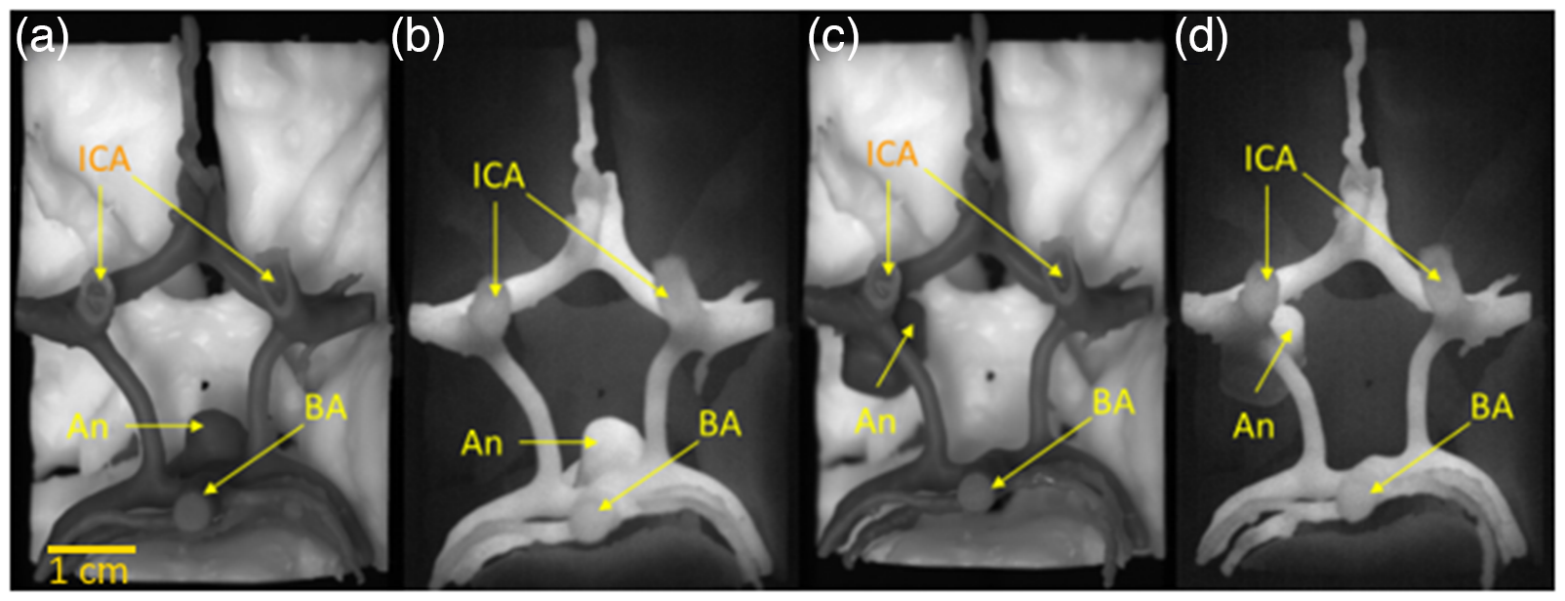
White light (a, c) and NIRF images (b, d) of partially assembled phantom with inferior view of circle of Willis region. Images show gray matter and brain stem with optic nerve components incorporating (a, b) BA aneurysm and (b, d) posterior communicator aneurysm. ICA, internal carotid artery; BA: basilar artery; and An, aneurysm.

**Fig. 13:**
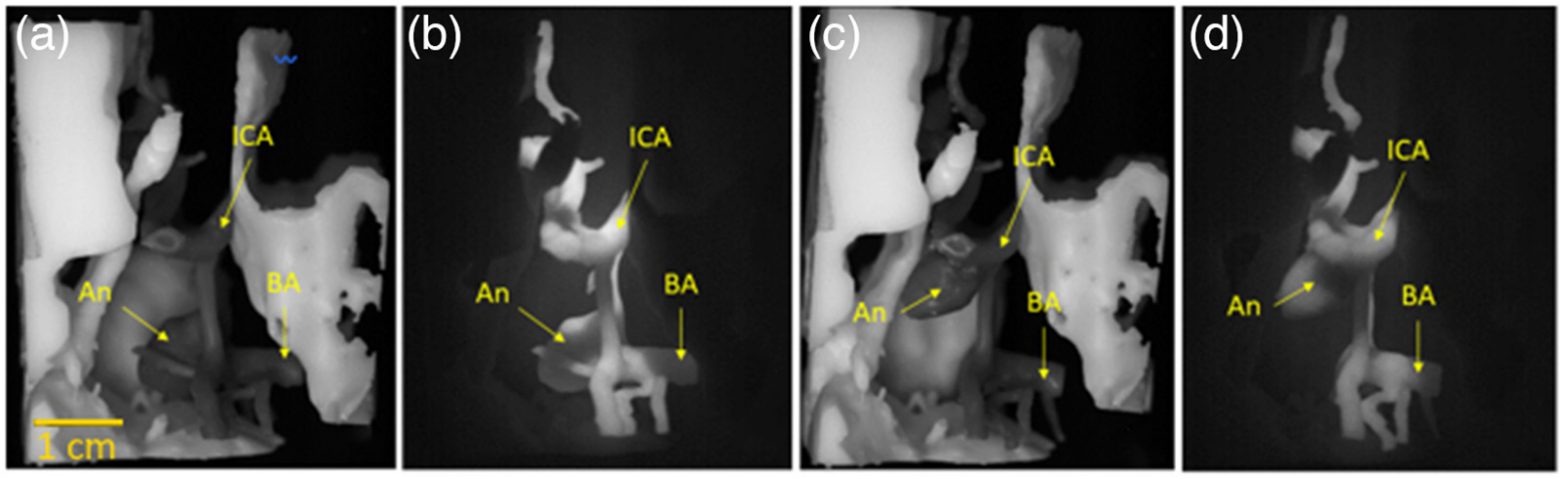
Lateral view white light (a, c) and fluorescence (b, d) images of final assembled aneurysm phantom – comprising circle of Willis, gray matter, brain stem, optic nerve and BA aneurysm (a, b) and posterior communicator aneurysm (c, d). ICA, internal carotid artery; BA: basilar artery; and An: aneurysm.

NIRF and white light images of the final circle of Willis components can be seen in Fig. 10. Fluorescence images of the vascular components indicate that the aneurysm and surrounding arteries are clearly visible against a black background. No significant problems were encountered during removal of support material from the dura mater and gray matter models. The dura mater model is shown in Fig. 11 both before and after support material removal. Because the gray matter was printed as two components divided by the longitudinal fissure, the distance between the two lobes was recorded before the support material was removed. It was particularly challenging to remove support material from the brain stem and optic nerve models due to their small, fragile features. Thin attachment structures were broken and the cranial nerves I, III, and VI cracked in several locations during post-processing. At the end of the support removal step only cranial nerve II remained intact. The cranial nerve and brainstem component can be seen before and after support material removal [Figs. 11(b), 11(c)]. When the non-fluorescent and fluorescent elements are assembled into a final phantom (Figs. 12 and 13), the fluorescent vasculature is still distinguishable from other tissue.

**Fig. 14:**
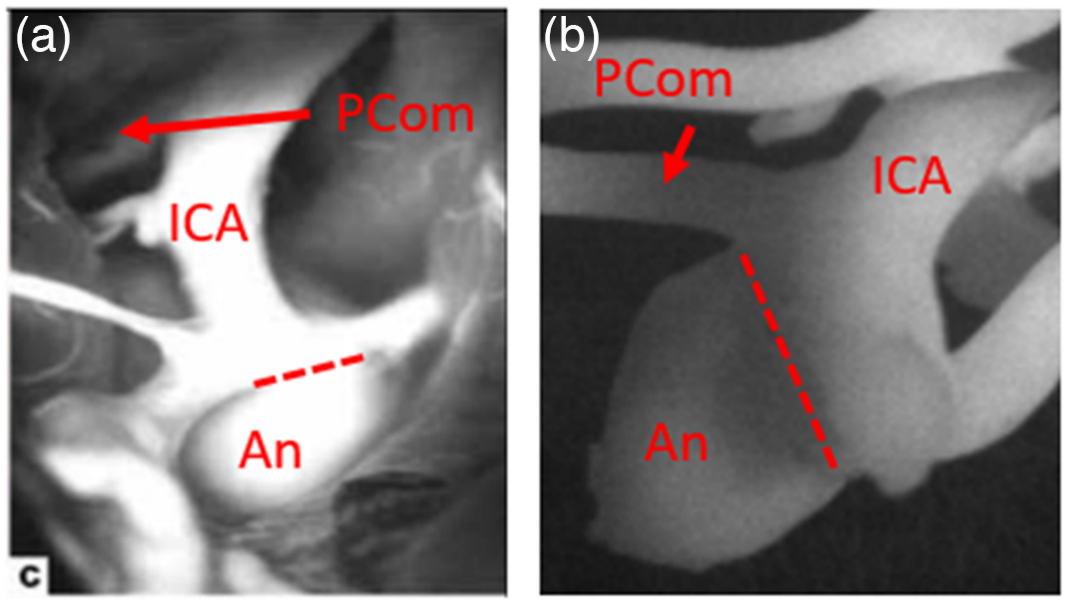
(a) NIRF images of a left anterior choroidal artery (AChA) aneurysm *in vivo* from Li et al.^35^ (reproduced with permission) and (b) our PCom aneurysm phantom (internal carotid artery: ICA). In each image, the dome of the aneurysm is labeled with the letters “An,” and the neck of the aneurysm is indicated with a dashed line.

A comparison of our phantom with an intraoperative NIRF image of an aneurysm is provided in Figure 14. Although the two aneurysms are not in the same location, these images illustrate the degree of realism that can be achieved with 3D-printed fluorescent phantoms. Critical landmarks such as the internal carotid artery (ICA) and the neck of aneurysm can clearly be distinguished in the clinical and phantom images.

Overall, our results indicate the potential for fabricating biomimetic phantoms for performance assessment and standardized comparison of NIRF imaging devices intended for specific clinical applications. With further refinement, our aneurysm model may be used for objective or subjective evaluation of surgical microscopes or other emerging clinical imaging systems. Additionally, there is a potential for these types of models to be used as surgical training tools to more rapidly familiarize clinicians with advanced optical technologies.

While this work has accomplished significant strides towards our objective, additional tasks and challenges remain. The printer used in this study is relatively inexpensive and provides a high degree of flexibility to adjust optical properties through the addition of absorbing, scattering and fluorescent constituents. However, printing of vessels or cranial nerves with diameters of less than 1 mm often results in fragile components that can break during removal of support material and phantom assembly. We are investigating alternate materials and expect that future devices and materials may provide improvements. Additionally, phantom colors (in white light imaging) can be altered using proprietary resin dyes. Developing a custom resin that appears more realistic would enhance phantom viability as a surgical training tool. Also, matching the optical properties of the non-fluorescent parts of the phantom to the optical properties of their respective tissues could better simulate the clinical environment.

## 4 Conclusion

Towards the objective of clinically relevant performance test methods for contrast-enhanced fluorescence imaging, we have developed tissue-simulating phantoms exhibiting complex cerebral morphology that have direct relevance to neurosurgery. The reproduction of common intracerebral aneurysms within the circle of Willis was achieved through the use of publicly available MRI-derived digital models. By developing a custom 3D-printed turbid resin doped with IR-125, it was possible to replicate fluorescence signals seen in arteries during ICG angiography procedures. Preliminary evidence of high geometric accuracy in our 3D-printed models was also obtained. These findings indicate that with some additional modifications, biomimetic neurosurgical phantoms for fluorescence imaging may emerge as a practical tool for device development and evaluation in the near future.
